# Geometry Does Impact on the Plane Strain Directions of the Human Left Ventricle, Irrespective of Disease

**DOI:** 10.3390/jcdd9110393

**Published:** 2022-11-15

**Authors:** Paolo Piras, Ivan Colorado-Cervantes, Paola Nardinocchi, Stefano Gabriele, Valerio Varano, Giuseppe Esposito, Luciano Teresi, Concetta Torromeo, Paolo Emilio Puddu

**Affiliations:** 1 Dipartimento di Ingegneria Strutturale e Geotecnica, Sapienza Università di Roma, Via Eudossiana n.18, 00184 Rome, Italy; 2 Dipartimento di Matematica e Fisica, Università degli Studi Roma Tre, Via della Vasca Navale 84, 00146 Rome, Italy; 3 Dipartimento di Architettura, Università degli Studi Roma Tre, Via Aldo Manuzio 72, 00153 Rome, Italy; 4 Department of Advanced Biomedical Sciences, University Federico II, 80138 Naples, Italy; 5 Interventional Cardiology Unit, De Gasperis Cardio Center, Niguarda Hospital, 20162 Milan, Italy; 6 Dipartimento di Scienze Cardiovascolari, Respiratorie, Nefrologiche, Anestesiologiche e Geriatriche, Sapienza Università di Roma, 00161 Rome, Italy; 7 Associazione per la Ricerca Cardiologica, Via Savoia 78, 00198 Rome, Italy; 8 Unité EA 4650, Signalisation, électrophysiologie et imagerie des lésions d’ischémie reperfusion myocardique, Université de Caen Normandie, 14000 Caen, France

**Keywords:** strain directions, tangent plane, circumferential direction, epicardium, endocardium, deformation tensor

## Abstract

The directions of primary strain lines of local deformation in Epicardial and Endocardial layers have been the subject of debate in recent years. Different methods led to different conclusions and a complete assessment of strain direction patterns in large and variable (in terms of pathology) cohorts of healthy and diseased patients is still lacking. Here, we use local deformation tensors in order to evaluate the angle of strain lines with respect to the horizontal circumferential direction in both Epi- and Endo-layers. We evaluated this on a large group of 193 subjects including 82 healthy control and 111 patients belonging to a great variety of pathological conditions. We found that Epicardial strain lines obliquely directed while those of Endocardium are almost circumferential. This result occurs irrespective of pathological condition. We propose that the geometric vinculum characterizing Endocardium and Epicardium in terms of different lever arm length and orientation of muscular fibers during contraction inescapably requires Endocardial strain lines to be circumferentially oriented and this is corroborated by experimental results. Further investigations on transmural structure of myocytes could couple results presented here in order to furnish additional experimental explanations.

## 1. Introduction

The complex architecture of the myocardium plays an important role in determining different cardiac functions [1,2,3,4,5] and significantly influences the presence of electromechanical patterns [6] and remodeling processes [7]. Its study and characterization are important not only for their potential use in the diagnosis and treatment of cardiac diseases [8] but also for the design of tissue-engineered grafts for myocardial repair [9]. Although myocardial architecture has been extensively investigated, there is no widespread agreement about its three-dimensional (3D) arrangement [10]. The most accepted theories describe the myocardium as a single muscular band wrapped in a helical pattern [11] or as a continuum of nested lamellae [12] but are still strongly questioned. Furthermore, other research emphasizes the ultrastructure of the myocardium, that is the 3D arrangement of the myocytes, and show experimental evidence of branching among individual myocytes [13] and of the existence of interconnected myocyte pathways through the myocardium [14]. This lack of consensus has led to a general description of the myocardium as a continuum made of contractile fibers, whose orientation varies transmurally. This architecture suggests that the fibers are the main load-bearing constituents, and that the principal strains should be oriented in the same direction of the fibers; moreover, longitudinal and circumferential strains are commonly used in the assessment of myocardial contractility.

Recent attempts have been made to answer this question, aided by 3D echocardiographic measurements. Pedrizzetti et al. [15] explored the idea of the use of the plane stress Principal Strain Lines (heretofore Primary Strain Directions, PSD) as a basis for the study of anisotropic materials, applying this concept to the heart, and showed that cardiac contraction occurred along functional paths suggesting a potential clinical significance. It is crucial to anticipate now that PSD discussed here are plane stress PSD, i.e., they belong to the local plane stress tangent to the surface of epicardium or endocardium and thus leading to two strain directions lying on that plane (see below).

Gabriele et al. [15], with a different computational protocol with regard to Pedrizzetti et al. [14], showed that PSD at the endocardium and epicardium are essentially circumferentially and obliquely disposed, respectively, thus suggesting that the PSD at the endocardium do not match with the supposed orientation of the muscle fibers. Their results have been reaffirmed in experimental and computational studies [16,17], concluding that PSD on the endocardial surface identify the lines which stiffen the Left Ventricle (LV) when it attains the highest pressure, making them the most important contributors to the volume change of the LV and a reliable indicator of contractility; conversely, at the epicardium, they can be used as a clue for the anatomical fiber orientation. This pattern appears in simulated data and was confirmed on a small group of real subjects [18]. Here, we extend the investigation presented in [18] to a larger sample of real cases consisting of 193 individuals composed of 82 healthy subjects and 111 patients belonging to 11 different clinical conditions including both volume and pressure overload pathologies.

**Figure 1:**
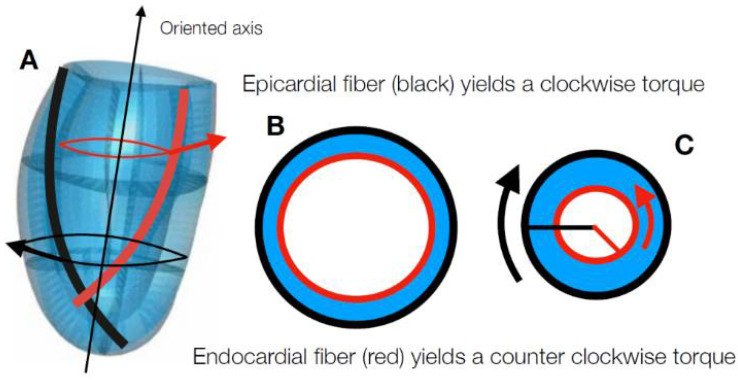
Cartoon showing the torque generation in LV muscular wall. (**A**) Epicardial fibers (black) have a left-handed helical pattern, while the endocardial ones (red) have a right-handed helical pattern. As a consequence, the hoop component of the contraction generates a torque around the vertical axis of the LV: epicardial fibers yield a clockwise torque, while the endocardial ones yield a counterclockwise torque. (**B**) Cross section of the LV at diastole, when contraction is null. (**C**) Cross section of the LV at systole, when contraction is high. If the contractile force is equal in the epicardial and endocardial fibers, the epicardial torque (black) is larger than the endocardial one (red), being different from the lever arm.

We measure the PSD angle with regard to the circumferential direction of the PSD projected tangentially to the Endo- or Epi- surfaces. The tangent plane is computed with regard to Endo- or Epi- local curvature following the critical suggestion made by [10]. PSD were computed by using landmark data acquired with 3D Speckle Tracking Echocardiography (3D-STE). We highlight here that we gave a landmark-wise representation of the strains which retains all the values computed with our data, as performed in [19]; there, it was suggested that the 16 sector-averages of the strains, usually adopted in many cardiological studies, are not enough for a fine assessment of deformation and motion of the LV. The first objective pursued in this study is to find strong experimental evidence of the hypothesis presented previously in earlier works [16,17,18,19] showing that PSD at the Endocardium tend to follow a circumferential direction while at the Epicardium they run obliquely and are closely oriented to the hypothesized anatomical fiber direction. Second, we want to propose a potential mechanistic explanation of why this happens. We hypothesize that the different PSD orientation at Endo- or Epi- depends on the particular geometric relationship of the Endocardium and Epicardium and on the initial fiber orientation assumed to follow inverse patterns at the undeformed state [10]. We propose that the larger curvature of the Endocardium and the smaller one of the Epicardium represent a geometric vinculum that constrains endocardial PSD to be almost circumferential in systole. The two layers can be thought of as two concentrical semi-ellipsoids that, under myocytes tightening, contract and rotate in opposite directions. However, the lever arm of the Endocardium is smaller with regard to the Epicardium and this causes its impossibility to maintain oblique direction. This model is complicated by the fact that endocardial and epicardial fibers, assumed to be oriented in opposite directions at starting undeformed state (diastole) are in continuity between each other along the myocardial wall and the very nature of fiber disposition along the transmural wall is a matter of controversy. Figure 1 shows, platonically, this notion as well as the opposite rotation of fibers during contraction.

## 2. Materials and Methods

### 2.1. Subjects

The study was performed under the approval of the review board of “Dipartimento di Scienze Cardiovascolari, Respiratorie, Nefrologiche, Anestesiologiche e Geriatriche, Sapienza Università di Roma”, and in accordance with the ethical guidelines of the Declaration of Helsinki. Written informed consent was obtained from each subject. All patients were enrolled to echocardiographic examinations by an Aplio–Artida ultrasound system (Toshiba Medical Systems Co., Tokyo, Japan) thanks to an official research and development agreement between the Dipartimento di Scienze Cardiovascolari, Respiratorie, Nefrologiche Anestesiologiche e Geriatriche, Sapienza Università di Roma and Toshiba Medical System Europe (Zoetermeer, The Netherlands). The dataset consists of 193 subjects; they are divided into 12 categories, see Table 1. Table 2 shows demographic parameters of the population under study. Clinical diagnoses were made by an expert cardiologist on the basis of genetics, echocardiographic imaging and physiological parameters.

As we used a part of the same dataset used in [20], we take for granted the reproducibility analysis presented there, including intra- and inter- observer variation. We also added to the above-mentioned reproducibility analysis for the following: we randomly selected a sub-sample of 14 individuals (7 Controls and 7 subjects affected by genetically determined Hypertrophic Cardiomyopathy) and we reconstructed them twice under the same hand (GE). As tensor estimation uses initial landmarks positions we used cartesian coordinates of systolic states of per-individual repeated acquisition in Bland–Altmann plots and in Lin’s concordance correlation coefficient (CCC) for agreement on a continuous measure.

### 2.2. Strains, Tensors, Strain Directions and More

**Figure 2:**
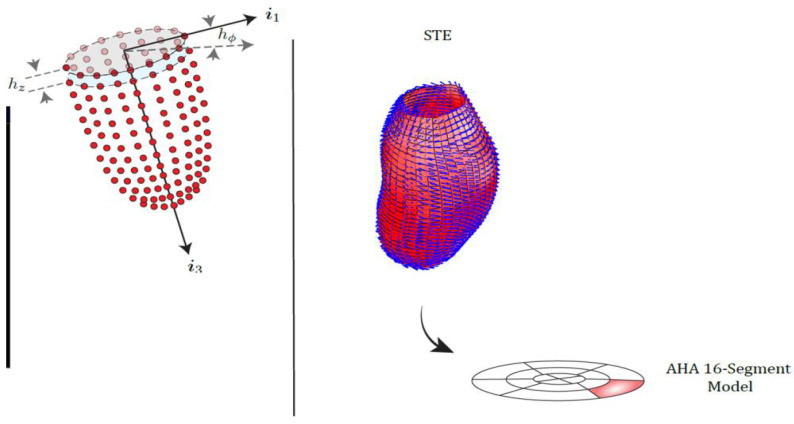
The 3D-STE can track the position of thousands of landmarks during the heart’s cycle. (**Left Panel**) Left: Typical 3D-STE landmarks output, i.e. the discretization of LV surfaces using the position of the landmarks tracked by the 3D-STE; each LV surface is segmented in 36 planes along the axial direction (base to apex); planes are equally divided into 36 sections along hoop direction. (**Right Panel**) Top: Example of PSD patterns as evaluated by using the original 3D-STE data. Bottom: PSD orientations and PSs are displayed on the American Heart Association (AHA) bull’s-eye 16-segment model. Modified from [19].

We analyzed 3D-STE data following the method of principal strain analysis proposed in [16]. The data set of each subject contains the positions of 1296 landmarks (36 parallels each with 36 landmarks) for both the endocardial and the epicardial surfaces of the LV, at diastole and end-systole. On Figure 2, the left panel shows the data as outputted by the 3D-STE echocardiographer;the right panel shows a typical PSD pattern and its representation in the American Heart Association (AHA) bull’s-eye 16-segment model. At any landmark location, we compute the angle α*_p_* that is measured as the deviation from the locally tangent horizontal direction; this was done at both Endocardium and Epicardium.

Under 3D-STE procedure the landmarks at the diastole and end of the systole are treated as anatomically homologous and tracked in time; thus, we can use the positions of these landmarks to compute local deformation in terms of strain tensors. The 3D-STE procedure is the application of pattern-matching technology to ultrasound cine data and is based on the tracking of the ‘speckles’ in a 3D volume; speckles are disturbances in ultrasounds caused by reflections in the ultrasound beam: each structure in the body has a unique speckle pattern that moves with tissue. A cubic template image 1 cm^3^ sized is created using a local myocardial region in the starting frame of the image data. It is worth noting that this does not mean that resolution is at most 1 cm; indeed, 1 cm^3^ is the dimension of the cubic template (in which the estimation point is centered) followed along different frames in the cardiac cycle, and not the distance between nearby estimation points to be followed in the same frame, which is approximately 0.25 cm. At any successive frame, the algorithm identifies the local speckle pattern that most closely matches the template. Temporal resolution is about 40 ms.

At this point it is crucial to clarify some points about deformation tensors when applied to a 3D shape such as a human left ventricle [21]. A full 3D deformation tensor **F** is a non-singular 3 × 3 matrix derived from the (infinitesimal) displacement field occurring between an undeformed state (source), a diastole in cardiological studies, and a deformed state, i.e., a target such as a systole. **F** also encodes an infinitesimal local rotation as **F** = **RU** with **R as** the rotation matrix from the polar decomposition on **F**. There are two main ways to compute **F**: (*i*) Continuously using the first derivative of an appropriate interpolant; this allows for the evaluation of **F** (reasonably) anywhere in the space. The interpolant computes the coefficients by taking the source’s and target’s landmarks; a convenient interpolant could be the Thin Plate Spline (Bookstein, 1989), as shown by [22]. (*ii*) Using a direct calculation on tetrahedra coordinates (in 3D when available) or triangle’s coordinates (in both 2D and 3D when available) or a first order neighborhood with regard to the evaluation point; using triangles in 3D or a plane-structured neighborhood leads to a singular 3 × 3 **F** matrix as it refers to a plane (see below).

The right Cauchy-Green symmetric deformation tensor **C** = **F^T^F** is symmetric and positive definite and can be used for computing **U** = $\sqrt{C}$ and for visualizing tensor behavior on the source configuration as they do not encode local rotation. We define **v_1_**, **v_2_** and **v_3_**, and ***λ*_1_**, ***λ*_2_** and ***λ*_3_** as the eigenvectors and eigenvalues, respectively, of **U**. One can use **v** and ***λ*** in order to depict deformation’ directions of a unitary circumference (in 2D or 3D either) or a sphere (in 3D), conveniently rescaled (for sake of visualization) if necessary, and the principal axes of the resulting ellipses (or ellipsoids in 3D). Planar ellipses can be also visualized in 3D in order to depict deformations of meshe’s triangles in the 3D space (see below). The principal axes identify the (reciprocally) orthogonal principal strain direction (SD) given by **v** while ***λ*** gives their magnitudes. As stated above, **C** does not encode rotation; thus U, **v** and ***λ*** must be used in order to map SD, and to depict deformation ellipses (in both 2D and 3D) or ellipsoids (in 3D), on the *source* shape; in order to depict this information on the target shape using SD encoded in **v**, they must be pre-multiplied by **F** in order to depict SD on the target configuration such as **v_y_** = **Fv**. This intrinsically encodes SD magnitudes. This procedure rotates **v** within the target according to **R** encoded in **F**. SD possesses an important deformational and mechanical meaning due to the results of specific forces such as in the left ventricle contraction [16,21,23,24]. ***λ****_i_* = 1 indicates no deformation, ***λ****_i_* > 1 indicates a deformation that produces an expansion (=tensile SD) along the direction of the corresponding SD, while ***λ****_i_* < 1 indicates a deformation that produces a compression (=compressive SD). The closer the ***λ****_i_* to 1, the smaller the deformation. This means that in case of ***λ****_i_* > 1 or ***λ****_i_* < 1, the direction of maximal deformation $\hat{\lambda }$ is dictated by the **v***_i_* corresponding to the ***λ****_i_* most distant from 1 such that $\hat{\lambda }$ = *argmax*(|1 − ***λ*|**). The corresponding direction of maximal deformation (either tensile or compressive) $\hat{v}$, on the source, or ${\hat{v}}_{y}$ on the target, is called primary strain direction (PSD).

Piras et al. [22] highlighted the differences between the full tensor F in 3D and its projection $\bar{F}$ on a plane. This plane is often represented by three points defining a single triangle in 3D in meshes equipped with triangulation connectivity matrix but it can be defined in arbitrary ways such as a first order neighborhood [16]. While F notifies the actual deformation occurring in the whole ambient space (infinitesimally), $\bar{F}$ is referred just to its projection on the plane. $\bar{F}$ is still a 3 × 3 matrix but it is singular and must be reduced to a dimension 2 × 2 by a change of basis thus obtaining $\bar{F}$*_p_* in order to extract numerical diagnostics. To $\bar{F}$ and $\bar{F}$*_p_* corresponds $\bar{C}$, $\bar{C}$*_p_*, $\bar{U}$ and $\bar{U}$*_p_* as well as ***λ****_p_*, **v***_p_*, ${\hat{\lambda }}_{p}$, $\hat{v}$*_p_* and ${\hat{v}}_{yp}.$

**Figure 3:**
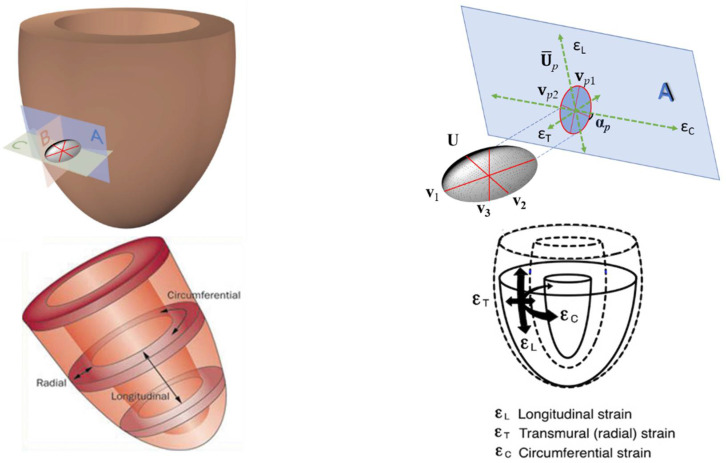
The relationships between full 3D tensor **U** (and its principal directions), its projection $\bar{U}$*_p_* on the local surface tangent plane and the classic radial, circumferential and longitudinal 3D-STE strains. Modified from [10].

At this point it is useful to recall the relationship between **U**, $\bar{U}$*_p_*, ***λ***, **v**, ***λ****_p_* and **v***_p_* with the traditional strains computed in the usual 3D-STE. Classical radial (ε_T_), longitudinal (ε_L_) and circumferential (ε_C_) strains can be thought as elongations/shortenings along anatomically homologous directions that parallelize the notion of homologous landmarks in modern shape analysis. However, while in the case of landmarks, the concept of homology is more easily spatially perceptible, in the case of a direction one is forced to look at it in the context of the whole physical body where this direction is defined. When speaking about the above-mentioned strains, these directions are dictated by platonic pathways (Figure 3 bottom left and right) oriented (*i*) towards the central axis of the left ventricle orthogonally to local tangent plane A (radial), (*ii*) along the left ventricle local circumference (circumferential) and (*iii*) orthogonally to the circumferential direction (longitudinal) on the ventricle’ tangent surface. The radial strain is usually computed considering the myocardial thickness change along the above-mentioned radial direction.

Both circumferential and longitudinal strain directions are contained in the A-plane, while the radial one is orthogonal to it and does not appear in the strains computed on it. Actually, in a real deformation, the sole object that describes entirely what happens locally is the full tensor **U** and its three SD **v_1_**, **v_2_** and **v_3_** (Figure 3 top left); if we decide to project it on the A-plane in order to see what happens on the local tangent surface, we use $\bar{U}$*_p_* and its two SD **v***_p_*_1_ and **v***_p_*_2_ that explains just the shadow of **U** on the A-plane. Moreover, if we add to the plane ε_L_, ε_C_ and ε_T_ (orthogonal to the A-plane) we see that they form angles with both **v***_p_*_1_ and **v***_p_*_2_ and **v_1_**, **v_2_** and **v_3_** collections. This means that evaluating local left ventricular deformation could be a very multifaceted operation and one should be aware of what specifically they are looking for when comparing cohorts of different categories.

In this paper, we face the behavior of the plane strain tensor $\bar{U}$*_p_* at the end-systole (the deformed state) with respect to the diastole (the undeformed state). $\bar{U}$*_p_* refers to stress and strains that are tangential to the epicardial or endocardial local surfaces and thus do not encode the radial dimension. Moreover, its SD are not projected along 3D-STE strains thus they are free to rotate on the A-plane. We want to evaluate the angle α*_p_* formed by PSD and the circumferential platonic direction (Figure 3 top right) in order to test the hypothesis that α*_p_* is (almost) invariably closer to 0 in the Endocardium than in the Epicardium.

**Figure 4:**
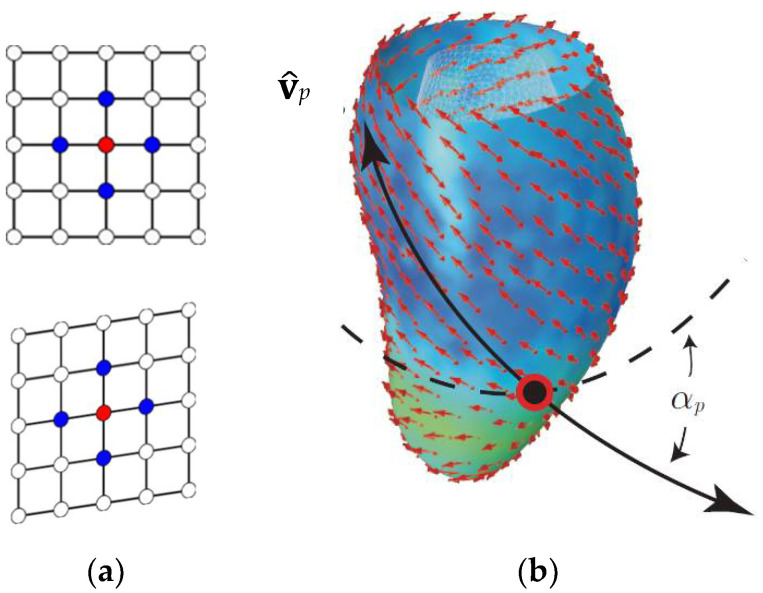
(**a**) First order neighborhood (in blue) relative to the evaluation point (in red) in a landmark-grid detail (either Endo- or Epi-) showed in the undeformed (diastole) and deformed state (systole). (**b**) Plane strain tensor $\bar{U}$*_p_* is calculated on the red point using the displacement vectors of the blue ones. The PSD $\hat{v}$*_p_* is then identified upon the eigen decomposition of $\bar{U}$*_p_*. Then, the angle α*_p_* is computed with regard to the circumferential direction (dotted line). Modified from [19].

Under our setting, the left ventricle structure is not treated as a cylinder and the curvature for the tangent plane computation is always local [10]. $\bar{U}$*_p_* was computed directly from the reciprocal position of landmarks in the diastole and systole, taking the first order neighborhood of each location, as shown in Figure 4 left panel. The procedure was previously applied and described in [16,17]. The eigen analysis of $\bar{U}$*_p,_* yields ***λ****_p_* and **v***_p_*. It is worth noting that in this experimental case, we always found the PSD ${\hat{\lambda }}_{p}$ = ${\hat{\lambda }}_{2p}$, corresponding to the deformation’ ellipse smaller axis. This means that the shortening occurring approximately along the circumferential direction is stronger than that occurring approximately along the longitudinal one.

### 2.3. Statistical Analysis and Visualization

#### 2.3.1. Epi-Endo Comparison

A procedure aimed at comparing the different mechanical behaviors of Epi- and Endocardium for each category must take into account two fundamental aspects: (1) as the angles are computed at each landmark, it should be considered that a simple ANOVA of distributions of angles is not adequate to correctly compare the Epi- and Endo-values. In fact, as each landmark in Epi- has its corresponding counterpart on Endo-, a paired test is more appropriate; (2) moreover, as our angles spans from −90° to 90°, for the purpose of testing Epi-Endo differences (in terms of deviation from horizontal tangent direction) it should be better to use their absolute values as the original ones are highly bimodally distributed. For these reasons we choose to adopt a nonparametric paired test, the Wilcoxon signed-rank test. For each of the 12 categories under study, each bearing *n* individuals, and for each of the 1296 landmarks of Epi- and Endo-, we performed the above-mentioned test on a vector with 2*n* values corresponding to landmark-specific individual absolute PSD angle values of the Epi- and Endo- for all individuals belonging to the *i*-th category and using a vector of Epi–Endo affiliation (thus 2*n* long) as the factor variable. We also computed the effect size following [25] that spans from 0 to 1.

#### 2.3.2. Control vs. Diseased Patients Comparison

In order to test the pervasiveness of the mechanical constraint underlying our working hypothesis depicted in Figure 1, we performed, landmark-wise, the comparison between PSD angle endo-epi absolute differences between healthy Controls and those of the other categories (calculated as Endo-Epi differences matching each landmark location). In this case, the Wilcoxon test should not be paired as different samples (i.e., Controls vs. Diseased) are compared for the same landmark location.

#### 2.3.3. PSD Angle Differences vs. Static Curvature Ratio

Actually, speaking about a larger or smaller radius means speaking about a larger or smaller curvature is what exactly represents the geometrical vinculum that would explain the orientation of PSD in Epi- or Endo- during systolic contraction. This vinculum is predicted to be present irrespective of the healthy or diseased condition in a beating heart.

Following the rationale illustrated by Figure 1, we correlated the curvature ratio between the two layers with differences between absolute Endo and absolute Epi α*_p_* values at each landmark’s location (per category and per landmarks median values). It is worth noticing here that the curvature is not calculated taking into consideration the deformation as this will lead to a circular logic being PSD computed upon systo–diastolic deformation. Instead, the curvature is calculated statically (in diastole or systole). Of the 36 circles each with 36 landmarks, we left out the last three circles, i.e., the final cap, as the curvature and PSD are more difficult to compute tangentially and bring a larger error. This does not weaken the validity of evidence. The curvature was estimated at each landmark location by considering the triangulation structure (connectivity) identifying Endo- or Epi- surfaces. It was calculated as Root Mean Square curvature (RMS), i.e., the square root of the sum of the two principal curvatures (k1 and k2) measured at each landmark location. Looking at Figure 1, one could argue that the 1/r with r the radius of Epi- or Endo- at each landmark location could be used but this could be applied to a cylindrical shape. Instead, as shown in Figure 2, the shape of a ventricle is not platonically cylindrical but irregular with concavities and convexities thus with a local curvature continuously varying along its surface. The use of local curvature (thus considering the connectivity between landmarks) provides a more realistic measure of the layer’s local curvature features. Under the hypothesis that a larger local curvature implies larger Endo–Epi differences in α*_p_*, we expect a negative relationship among differences between absolute-Endo and absolute-Epi α*_p_* values vs. Endo/Epi curvature ratio.

#### 2.3.4. Results Visualization

In order to depict results meaningfully, thus considering the spatial regionalization, the statistical significance, and the extent and direction of differences in α*_p_*, we used the bull’s eye 16-segment representation proposed by the AHA. While it could be considered too platonic with respect to the real 3D geometries, this representation offers a better immediate look at both the positions of landmarks in the LV wall and at their values, represented in color code. These plots refer to per-category median values computed at each landmark of the LV. Concerning the PSDs, besides the figures depicting the α*_p_* at Epi- and Endo- for each category, we added figures showing the significance of the differences in α*_p_* between Endo- and Epi- surfaces and between CNT and the other groups. In these latter figures, a color scale was used only for those landmarks where the associated statistical test was significant (significance level = 0.05); landmarks were colored in cyan when the test was not significant.

## 3. Results

Reproducibility analysis revealed a high rate of confidence between successive replicas (here the vector of ρ’ CCC metric: 0.992, 0.986, 0.995, 0.994, 0.997, 0.987, 0.996, 0.99, 0.995, 0.992, 0.99, 0.996, 0.995, 0.984). Supplementary Figure S1 shows the 14 Bland–Altman plots.

### 3.1. Epi-Endo Comparison

**Figure 5:**
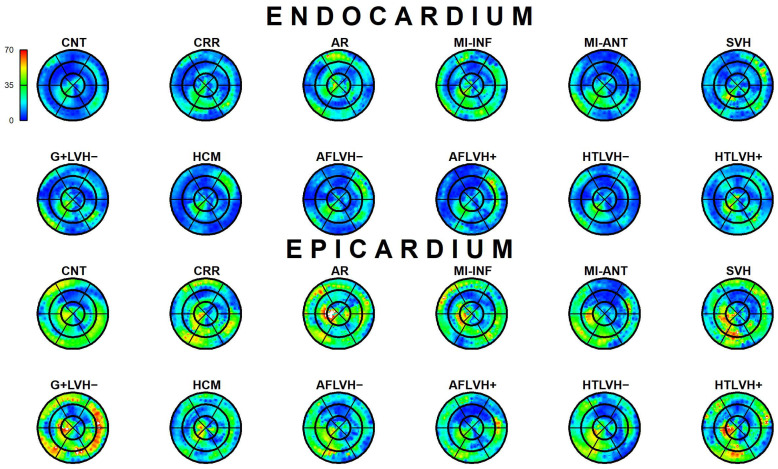
Per-category and per-landmark median values of α*_p_* absolute values in degrees for both Epi- and Endocardium. Regions are colored according to the color bar at top left. Abbreviations: Control healthy subjects (CNT), Cirrhosis (CRR), Aortic regurgitation (AR), Myocardial infarction of inferior wall (MI-INF), Myocardial infarction of anterior wall (MI-ANT), Mutation carriers for hypertrophic cardiomyopathy with LV hypertrophy (HCM), Secondary ventricular hypertrophy (SVH), Mutation carriers for hypertrophic cardiomyopathy without LV hypertrophy (G+LVH−), Atrial fibrillation without ventricular hypertrophy (AFLVH−), Atrial fibrillation with ventricular hypertrophy (AFLVH+), Hypertension without LV hypertrophy (HTLVH−), and Hypertension with LV hypertrophy (HTLVH+).

**Figure 6:**
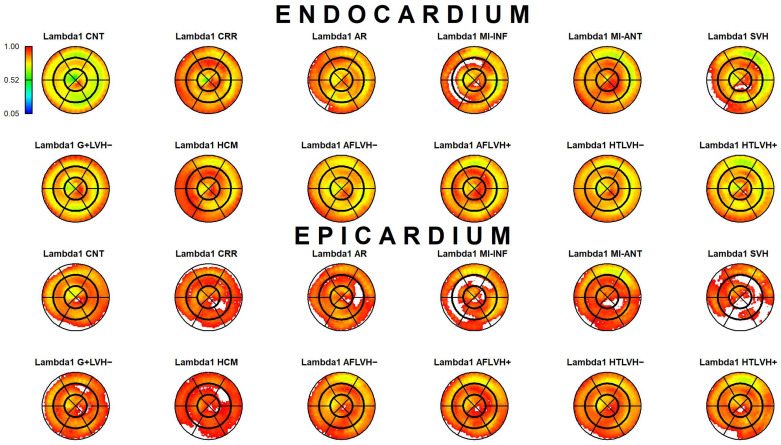
Per-category and per-landmark median values of ***λ***_**1***p*_ for both Epi- and Endocardium. Regions are colored according to the color bar at top left. Abbreviations: Control healthy subjects (CNT), Cirrhosis (CRR), Aortic regurgitation (AR), Myocardial infarction of inferior wall (MI-INF), Myocardial infarction of anterior wall (MI-ANT), Mutation carriers for hypertrophic cardiomyopathy with LV hypertrophy (HCM), Secondary ventricular hypertrophy (SVH), Mutation carriers for hypertrophic cardiomyopathy without LV hypertrophy (G+LVH−), Atrial fibrillation without ventricular hypertrophy (AFLVH−), Atrial fibrillation with ventricular hypertrophy (AFLVH+), Hypertension without LV hypertrophy (HTLVH−), and Hypertension with LV hypertrophy (HTLVH+).

**Figure 7:**
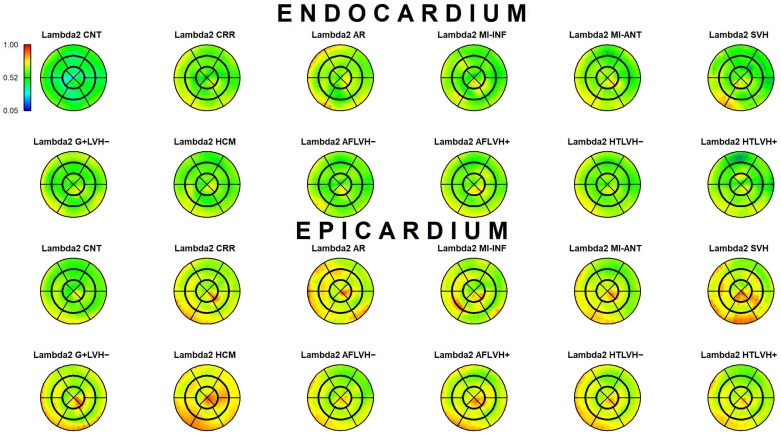
Per-category and per-landmark median values of ***λ***_**2***p*_ for both Epi- and Endocardium. Regions are colored according to the color bar at top left. Abbreviations: Control healthy subjects (CNT), Cirrhosis (CRR), Aortic regurgitation (AR), Myocardial infarction of inferior wall (MI-INF), Myocardial infarction of anterior wall (MI-ANT), Mutation carriers for hypertrophic cardiomyopathy with LV hypertrophy (HCM), Secondary ventricular hypertrophy (SVH), Mutation carriers for hypertrophic cardiomyopathy without LV hypertrophy (G+LVH−), Atrial fibrillation without ventricular hypertrophy (AFLVH−), Atrial fibrillation with ventricular hypertrophy (AFLVH+), Hypertension without LV hypertrophy (HTLVH−), and Hypertension with LV hypertrophy (HTLVH+).

**Figure 8:**
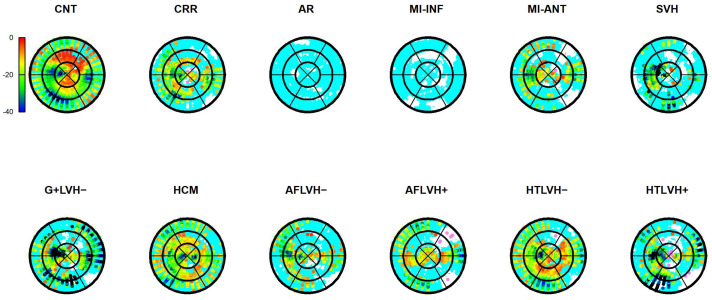
Per-category and per-landmark median values Δk measuring Endo-Epi α*_p_* absolute differences. For each category all landmark locations were subjected to the Wilcoxon paired nonparametric test (i.e., endo- vs. epi-). Regions are colored according to the color bar at top left; Δk < −40 are colored in black: Δk > 0 and with a significant test (very limited) are colored in violet; Δk with a non-significant test are colored in cyan if <0, in white if >0. Abbreviations: Control healthy subjects (CNT), Cirrhosis (CRR), Aortic regurgitation (AR), Myocardial infarction of inferior wall (MI-INF), Myocardial infarction of anterior wall (MI-ANT), Mutation carriers for hypertrophic cardiomyopathy with LV hypertrophy (HCM), Secondary ventricular hypertrophy (SVH), Mutation carriers for hypertrophic cardiomyopathy without LV hypertrophy (G+LVH−), Atrial fibrillation without ventricular hypertrophy (AFLVH−), Atrial fibrillation with ventricular hypertrophy (AFLVH+), Hypertension without LV hypertrophy (HTLVH−), and Hypertension with LV hypertrophy (HTLVH+).

The results of Epi-Endo comparison are illustrated in Figure 5. The bull’s eye plots refer to the per-category and per-landmark median of absolute α*_p_* values. The scientific rhetoric carried by this figure is actually at the limit of self-evidence: plane stress PSD in Endo- present an α*_p_* constantly smaller and closer to 0 with regard to Epicardium. This signal is constant among all categories analyzed in this study irrespective of disease condition. There is a little variation among pathologies with regard to the control but always under the notion of Endo α*_p_* < Epi α*_p_*. We then start from this observation to proceed with Epi–Endo statical comparison as well as for CNT versus the other categories. Figure 6 and Figure 7 show the per-landmark and per-category median values of ***λ*_1_***_p_* and ***λ*_2_***_p_*, respectively. As anticipated above, we always found both eigenvalues < 0 with few exceptions for ***λ*_1_***_p_* in some disease conditions. Moreover, as evident from the color code mapped in the bull’s eye plots, we found invariantly |1 − ***λ*_2_***_p_*| > |1 − ***λ*_1_***_p_*|. It is also interesting to note that, albeit the magnitude of strains is not the focus of the present paper, the control differs evidently from the other disease conditions (the control being more deformed), as previously highlighted in several studies regarding 3D-STE strains; the same cannot be said for α*_p_* on Figure 5. Figure 8 shows the Endo-Epi α*_p_* differences (Δk) subjected to the paired non-parametric test statistics (see Section 2). The key plot is that referred to the control group (CNT), it is not only the most represented cohort in terms of sample size (*n* = 82), but also because it represents the normal healthy condition. The other categories present very uneven sample sizes, and this inevitably affects the test’s power; for example, AR and MI-INF present only 4 individuals and the *p*-value should be considered with caution. Regions are colored according to the color bar on top left; Δk < −40 are colored in black; Δk > 0 and with a significant test (very limited) are colored in violet; Δk with a non-significant test are colored in cyan if <0, in white if >0. The first evidence we can observe is the fact that the violet color is nearly totally absent in all plots. This means that Endo-α*_p_* are ubiquitously more circumferential than Epi-α*_p_* independently from the disease condition. The second evidence is that CNT is nearly entirely occupied by negative values; this means that in the healthy control group Endo-α*_p_* are significantly more circumferential than Epi α*_p_*. In other categories, this is better observed in those categories represented by relatively larger sample sizes (HCM, HTLVH−). The presence of cyan color (i.e., non-significant test) is associated with those categories with very small sample sizes.

### 3.2. Control vs. Diseased Comparison

**Figure 9:**
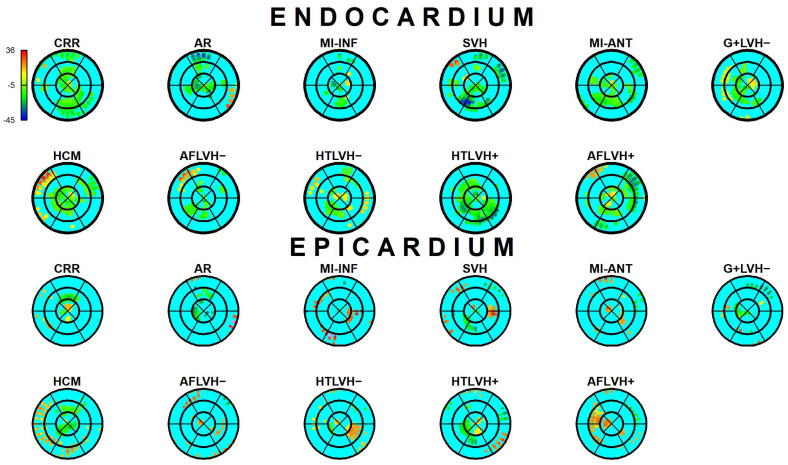
Differences ΔkCNT − Δkother between α*_p_* values of control group and other categories. Landmarks with no significant differences are colored in cyan. Abbreviations: Cirrhosis (CRR), Aortic regurgitation (AR), Myocardial infarction of inferior wall (MI-INF), Myocardial infarction of anterior wall (MI-ANT), Mutation carriers for hypertrophic cardiomyopathy with LV hypertrophy (HCM), Secondary ventricular hypertrophy (SVH), Mutation carriers for hypertrophic cardiomyopathy without LV hypertrophy (G+LVH−), Atrial fibrillation without ventricular hypertrophy (AFLVH−), Atrial fibrillation with ventricular hypertrophy (AFLVH+), Hypertension without LV hypertrophy (HTLVH−), and Hypertension with LV hypertrophy (HTLVH+).

The comparison between Δk in Control group and Δk of other categories is shown in Figure 9. As the cyan color indicates non-significance, we can see that there are no big diffreneces between healthy Control group and pathological categories.

### 3.3. α_p_ Differences vs. Static Curvature’ Ratio

**Figure 10:**
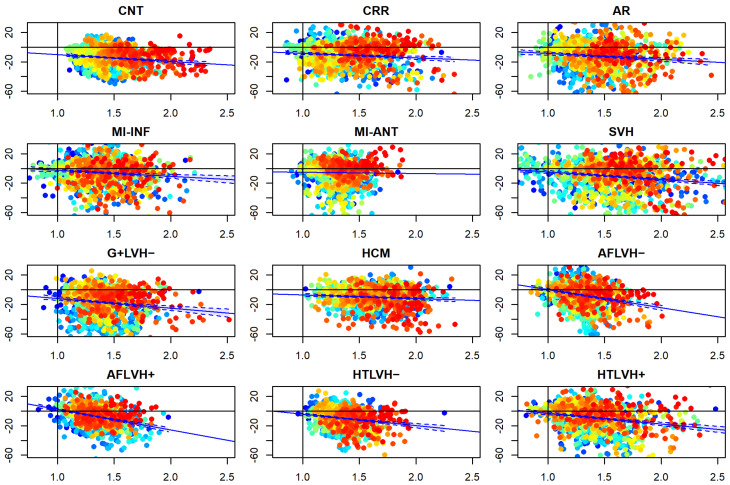
Relationship between Endo/Epi RMS static curvature ratio in systole (in abscissa) and Endo-Epi α*_p_* differences (per category and per-landmark median values). Blue lines represent regression lines with confidence intervals. Points are colored according to their affiliation to “parallels” in the context of LV topology shown in Figure 2: cyan at the base, yellow-green in the middle, red in the apex. The vertical lines at x = 1 and the horizontal one at y = 0 indicate the upper and lower limits (respectively) within which we expect to see the ranges of these variables. CNT behave consistently with this expectation (i.e., Endo/Epi RMS curvature ratio always >1 and α*_p_* differences always <1). The other categories do the same with a few exceptions.

**Figure 11:**
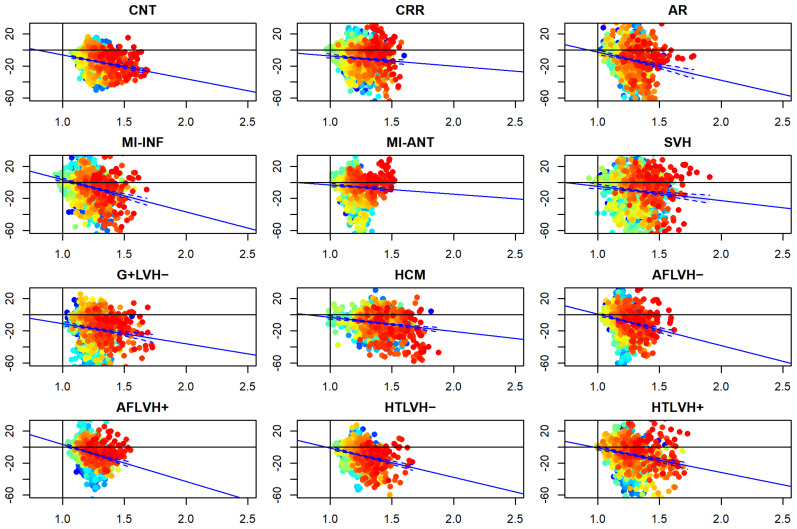
Relationship between Endo/Epi RMS static curvature ratio in diastole (in abscissa) and Endo-Epi α*_p_* differences (per category and per-landmark median values). Blue lines represent regression lines with confidence intervals. Points are colored according to their affiliation to “parallels” in the context of LV topology shown in Figure 2: cyan at the base, yellow-green in the middle, red in the apex. The vertical lines at x = 1 and the horizontal one y = 0 indicate the upper and lower limits (respectively) within which we expect to see the ranges of these variables. CNT behave consistently with this expectation (i.e., Endo/Epi RMS curvature ratio always >1 and α*_p_* differences always <1). The other categories do the same with a few exceptions.

Figure 10 and Figure 11 show the relationship among differences between absolute Endo and absolute Epi α*_p_* values regressed against the ratio of endo/epi RMS curvatures (per-category and per-landmark median values). Regression lines are also shown. Details about per-group regressions are shown in Table 3. The vertical lines at x = 1 and the horizontal one at y = 0 indicate the upper and lower limits (respectively) within which we expect to see the ranges of these variables. CNT behave consistently with this expectation (i.e., endo/epi RMS curvature ratio always >1 and α*_p_* absolute differences always <1). The other categories do the same with a few exceptions that we ascribe to small sample sizes that cause less robust per-landmark median values. Points are colored according to their affiliation to “parallels” in the context of LV topology shown in Figure 2: cyan at the base, yellow–green in the middle, red in the apex. The most important evidence is that both systole and diastole return similar results with negative β coefficients. Moreover, all categories behave consistently in the same manner: the larger the curvature ratio the larger absolute α*_p_* differences.

## 4. Discussion

We carried out a comparative study of PSD in human LVs. The statistical analysis performed allowed us to study the orientation variability of the PSD in different regions of the myocardial walls, distinguishing Epi- and Endo-layers, and how they differ in a quite large group of both healthy and diseased patients. Knowledge of myocardial architecture is essential to understand normal and abnormal cardiac function. Some viewpoints based on medical image processing have promoted the possible use of PSD to identify the fibrous architecture of the myocardium [15]. These proposals contrast with experimental evidence showing that the PSD may not coincide with the direction of the cardiac fibers on the Endocardium ([16,17,21], this study). Furthermore, it has been suggested that other structural mechanisms would play an important role in the determination of myocardial deformations. Detailed anatomical inspections have led to the hypothesis that obliquely bonded myocytes may be major contributors to structural reorganization when the cardiac tissue contracts [13,14]. This irregular structure, with respect to the main fiber orientation, would affect the strain patterns. Some previous studies already suggested the idea that the principal directions of deformation may not be related to the fiber direction, namely at the endocardium [26,27,28]. Myofiber arrangement within the myocardial mass is poorly known. In order to better understand the mechanism of contraction, it is important to have a detailed picture of this arrangement throughout the myocardial wall. It has been shown that, at the endocardium, the strain in the cross-fiber direction is larger than in the fiber direction. Moreover, it has been suggested that this may be related to the transmural interaction of myocardial layers with different fiber orientation [21]. In the same manner, Anderson et al. [13] and Smerup et al. [14] have hypothesized that myocytes obliquely branched from the main fiber lines form transverse structural elements that contribute to the three-dimensional reorganization and regional stabilization of the ventricular form; it was proposed that, when these lateral aggregates contract, they apply a load on the surrounding myocytes which is misaligned with respect to the main fiber axis, therefore affecting the local strain pattern and fiber shortening. Our results show that the PSD in the endocardium are oriented circumferentially irrespective of clinical condition, while in the epicardium they are directed in an oblique manner, close to the anatomy of the fiber. This is in good agreement with previous studies [11,14,15,20,28] and does not support previous statements and conclusions [15]. The statistical comparison of the endocardial and epicardial strain surfaces orientations showed that, in general, the orientation of the PSD is always larger at the epicardium. The statistical comparison between healthy and diseased patients did not show relevant differences. This means that the geometrical-physical vinculum occurring in a beating heart consisting in the larger curvature of endocardium relative to the epicardium cannot be violated even in presence of disease. This vinculum should thus be taken into consideration when interpreting and/or using information from 3D-echocardiography to be then applied to clinical interpretations. This can be explained by the larger curvature of the epicardial layer with regard to that of the endocardial one, see Figure 5. While during contraction the two layers would rotate in opposite directions according to their respective fiber orientation, the observed strain pattern comes by virtue of an unavoidable geometric restriction: the radius of the endocardium is always shorter than that of the epicardium. Thus, during contraction, despite the tendency to rotate according to fiber orientation of both layers, the endocardium is somehow dominated by the epicardium. Thus, the PSLs in this latter are roughly oriented along the fiber direction whereas the PSLs in the endocardium show a small deviation from the horizontal plane. This pattern appears in both simulated and real data [19]. It follows that PSD orientation should not be used as clinical indicators given their invariance in control and pathological subjects. On the contrary, looking at Figure 6 and Figure 7, one could look at strains even if in this case these strains are not coincident with those commonly used in 3D-STE investigations as they do not follow the classical homologous directions, i.e., longitudinal, radial and circumferential ones. The analysis of strains, however, was not the scope of the present study and it is for further research to explore their ability in distinguishing healthy from pathological conditions.

## 5. Limitation of the Study

Current echocardiographic technology is limited by low spatial and temporal resolution. Consequently, the information extracted from it is expected to be affected as well. Motion and shape can reveal useful information in terms of cardiac efficiency. The 3D-STE procedure is just one of the new technologies allowing the measurement of the complex multidimensional deformation of the heart [29,30]. Despite its good spatial and temporal resolution, it has been evidenced that this technique suffers from reduced accuracy in areas of low image quality [31]. Furthermore, despite the reproducibility analysis in [20], we are aware that some of the settings during the acquisition procedure are very subjective, depending on the abilities of the machine operator to highlight the regions of interest. However, although the principal strain analysis is not adequate to identify cardiac fiber architecture, it is a reliable and useful method to visualize strain and strain orientation patterns. This information, coupled with other methodologies, may be helpful to patient-specific medical diagnosis [32,33]. Other pathologies could be included in the present study including pathology of the right ventricle, such as tetralogy of Fallot. Unfortunately, our research group does not benefit anymore from the special agreement with Toshiba that allowed the use of the Aplio-Artida echocardiographer for about five years. Future opportunities will allow us to expand the pathology panel presented here to the right heart in order to extend to it the approaches used here.

## Figures and Tables

**Table 1:** Subjects categories.

Code	Description	N
CNT	control healthy subjects	82
CRR	cirrhosis	13
AR	aortic regurgitation	4
MI-INF	myocardial infarction of inferior wall	4
MI-ANT	myocardial infarction of anterior wall	11
SVH	secondary ventricular hypertrophy	7
G+LVH−	mutation carriers for hypertrophy without LV hypertrophy	7
HCM	mutation carriers for hypertrophy with LV hypertrophy	18
AFLVH−	atrial fibrillation without ventricular hypertrophy	9
AFLVH+	atrial fibrillation with ventricular hypertrophy	11
HTLVH−	hypertension without LV hypertrophy	18
HTLVH+	hypertension with LV hypertrophy	9

**Table 2:** Demographic parameters for the categories under study here.

Group\Variables	CNT N = 82		CRR N = 13		AR N = 4		MI-INF N = 4		MI-ANT N = 11		SVH N = 7		G+LVH− N = 7		HCM N = 18		AFLVH− N = 9		AFLVH+ N = 11		HTLVH− N = 18		HTLVH+ N = 9	
Sex (M/F)	48/34	*n* = 82	11/2	*n* = 13	2/2	*n* = 4	3/1	*n* = 4	10/1	*n* = 11	6/1	*n* = 7	3/4	*n* = 7	12/6	*n* = 18	8/1	*n* = 9	6/5	*n* = 11	10/8	*n* = 18	8/1	*n* = 9
	Mean ± sd	*n*	Mean ± sd	*n*	Mean ± sd	*n*	Mean ± sd	*n*	Mean ± sd	*n*	Mean ± sd	*n*	Mean ± sd	*n*	Mean ± sd	*n*	Mean ± sd	*n*	Mean ± sd	*n*	Mean ± sd	*n*	Mean ± sd	*n*
Age, years	38.531 ± 7.877	*n* = 81	57.077 ± 6.689	*n* = 13	61 ± 22.316	*n* = 4	58.25 ± 8.382	*n* = 4	60 ± 9.274	*n* = 11	57.286 ± 12.645	*n* = 7	45.143 ± 12.812	*n* = 7	48.5 ± 13.544	*n* = 18	59.778 ± 13.8	*n* = 9	71.636 ± 3.957	*n* = 11	56.389 ± 7.578	*n* = 18	57.667 ± 10.186	*n* = 9
Ejection Fraction, %	54.82 ± 8.679	*n* = 82	54.435 ± 7.321	*n* = 13	53.081 ± 9.729	*n* = 4	59.858 ± 6.912	*n* = 4	52.023 ± 5.123	*n* = 11	51.896 ± 9.184	*n* = 7	59.061 ± 3.598	*n* = 7	58.653 ± 7.828	*n* = 18	54.143 ± 6.921	*n* = 9	56.399 ± 7.5	*n* = 11	55.643 ± 10.143	*n* = 18	56.357 ± 3.921	*n* = 9
Weight, kg	71.409 ± 12.27	*n* = 66	71.385 ± 10.389	*n* = 13	64.25 ± 15.521	*n* = 4	83.25 ± 18.392	*n* = 4	81.909 ± 9.235	*n* = 11	76.286 ± 6.473	*n* = 7	72 ± 13.416	*n* = 7	76.111 ± 14.768	*n* = 18	85.444 ± 10.039	*n* = 9	74.455 ± 13.299	*n* = 11	75.944 ± 9.716	*n* = 18	80.556 ± 10.477	*n* = 9
BSA, m^2^	1.841 ± 0.193	*n* = 66	1.831 ± 0.157	*n* = 13	1.663 ± 0.216	*n* = 4	1.94 ± 0.244	*n* = 4	2.06 ± 0.111	*n* = 6	1.853 ± 0.065	*n* = 7	1.813 ± 0.166	*n* = 7	1.869 ± 0.175	*n* = 15	2.001 ± 0.203	*n* = 7	1.908 ± 0.175	*n* = 4	1.874 ± 0.155	*n* = 18	1.944 ± 0.15	*n* = 9
BMI, Kg/m^2^	23.909 ± 3.091	*n* = 66	24.103 ± 2.67	*n* = 13	23.208 ± 1.461	*n* = 4	29.105 ± 3.221	*n* = 4	26.666 ± 2.327	*n* = 11	27.396 ± 3.694	*n* = 7	25.993 ± 4.569	*n* = 7	25.869 ± 3.9	*n* = 18	27.437 ± 2.178	*n* = 9	25.985 ± 3.97	*n* = 11	26.447 ± 2.938	*n* = 18	27.039 ± 2.547	*n* = 9
Interv. Septum, mm	8.197 ± 1.449	*n* = 66	10.154 ± 1.345	*n* = 13	10.25 ± 2.5	*n* = 4	10.5 ± 2.082	*n* = 4	10.091 ± 1.375	*n* = 11	9.286 ± 2.289	*n* = 7	9.571 ± 1.618	*n* = 7	18 ± 4.256	*n* = 18	9.444 ± 1.81	*n* = 9	11.636 ± 2.111	*n* = 11	9.444 ± 1.542	*n* = 18	11.667 ± 1.225	*n* = 9
PAS, mmHg	116.406 ± 9.614	*n* = 64	125.615 ± 9.124	*n* = 13	118.75 ± 6.292	*n* = 4	121.25 ± 10.308	*n* = 4	114.091 ± 8.006	*n* = 11	134.286 ± 11.701	*n* = 7	122.857 ± 4.88	*n* = 7	123.824 ± 6.002	*n* = 17	127.778 ± 11.756	*n* = 9	128.182 ± 10.787	*n* = 11	129.722 ± 8.309	*n* = 18	130 ± 11.18	*n* = 9
PAD, mmHg	73.984 ± 7.724	*n* = 64	77.538 ± 8.038	*n* = 13	70 ± 8.165	*n* = 4	75 ± 9.129	*n* = 4	70 ± 8.062	*n* = 11	84.286 ± 4.499	*n* = 7	75.714 ± 5.345	*n* = 7	78.235 ± 5.574	*n* = 17	79.444 ± 3.909	*n* = 9	75.455 ± 5.222	*n* = 11	82.778 ± 7.117	*n* = 18	81.667 ± 7.906	*n* = 9

“N” indicates the individuals belonging to each category, while “*n*” indicates the sample size excluding case wise missing data for each variable. Abbreviations: Control healthy subjects (CNT), Cirrhosis (CRR), Aortic regurgitation (AR), Myocardial infarction of inferior wall (MI-INF), Myocardial infarction of anterior wall (MI-ANT), Secondary ventricular hypertrophy (SVH), Mutation carriers for hypertrophic cardiomyopathy without LV hypertrophy (G+LVH−), Mutation carriers for hypertrophic cardiomyopathy with LV hypertrophy (HCM), Atrial fibrillation without ventricular hypertrophy (AFLVH−), Atrial fibrillation with ventricular hypertrophy (AFLVH+), Hypertension without LV hypertrophy (HTLVH−), and Hypertension with LV hypertrophy (HTLVH+).

**Table 3:** Details of per-group regressions between Endo/Epi RMS curvature ratio vs. α*_p_* differences (per category and per-landmark median values). Results for systole and diastole are shown.

Group	Intercept	*p*-Value_Intercept	Beta	*p*-Value_Beta	R_Squared
		Systole			
CNT	−0.607	0.817	−9.427	7.57 × 10^−8^	0.024
CRR	−2.112	0.391	−6.164	3.24 × 10^−4^	0.011
AR	−0.691	0.822	−8.018	1.25 × 10^−4^	0.012
MI-INF	5.708	0.053	−8.185	9.51 × 10^−5^	0.013
SVH	−3.593	0.342	−1.579	0.576	0
MI-ANT	5.647	0.02	−10.307	1.08 × 10^−11^	0.038
G+LVH−	1.467	0.691	−13.208	2.81 × 10^−7^	0.022
HCM	−2.248	0.394	−4.833	0.003	0.007
AFLVH−	24.488	1.76 × 10^−13^	−24.431	3.37 × 10^−22^	0.076
HTLVH−	29.934	9.26 × 10^−20^	−27.755	5.87 × 10^−29^	0.1
HTLVH+	11.208	4.00 × 10^−4^	−15.447	1.65 × 10^−11^	0.038
AFLVH+	11.592	2.64 × 10^−5^	−14.542	4.19 × 10^−14^	0.047
		Diastole			
CNT	23.409	1.15 × 10^−6^	−29.695	3.7 × 10^−15^	0.051
CRR	5.217	0.333	−12.75	0.003	0.007
AR	32.119	6.05 × 10^−6^	−34.933	4.1 × 10^−10^	0.032
MI-INF	43.368	1.88 × 10^−14^	−40.097	4.7 × 10^−8^	0.061
SVH	8.12	0.172	−11.331	0.02	0.005
MI-ANT	12.818	0.025	−17.726	4.4 × 10^−5^	0.014
G+LVH−	14.257	0.03	−25.162	1.4 × 10^−6^	0.019
HCM	14.313	2.18 × 10^−4^	−17.576	3.6 × 10^−10^	0.033
AFLVH−	39.764	2.81 × 10^−10^	−39.072	5.3 × 10^−14^	0.047
HTLVH−	49.612	7.53 × 10^−16^	−46.261	5.1 × 10^−20^	0.068
HTLVH+	35.213	5.54 × 10^−12^	−36.41	9.9 × 10^−19^	0.064
AFLVH+	29.519	7.87 × 10^−8^	−30.599	2.3 × 10^−12^	0.041

## Data Availability

Data can be obtained from the authors upon request.

## Supplementary Materials

The following supporting information can be downloaded at: https://www.mdpi.com/article/10.3390/jcdd9110393/s1, Figure S1: The 14 Bland–Altman plots.
