# Supplementary figures and images for: Geometry Does Impact on the Plane Strain Directions of the Human Left Ventricle, Irrespective of Disease
Source: J Cardiovasc Dev Dis. 2022 Nov 15;9(11):393. doi: 10.3390/jcdd9110393 (PMC9692678; doi:10.3390/jcdd9110393)

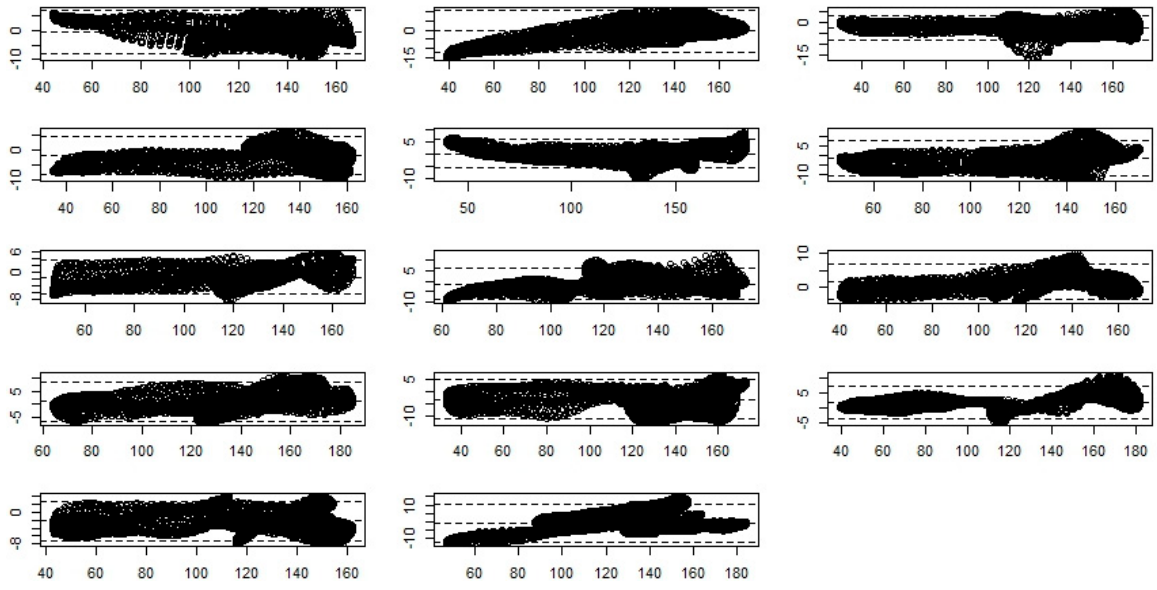

Figure S1. The 14 Bland–Altman plots.

Supplement: Supplementary file 1 [file jcdd-09-00393-s001.zip › jcdd-1964337-supplementary.pdf]
